# Maximum Neighborhood Margin Discriminant Projection for Classification

**DOI:** 10.1155/2014/186749

**Published:** 2014-02-20

**Authors:** Jianping Gou, Yongzhao Zhan, Min Wan, Xiangjun Shen, Jinfu Chen, Lan Du

**Affiliations:** ^1^School of Computer Science and Telecommunication Engineering, JiangSu University, ZhenJiang, JiangSu 212013, China; ^2^School of Mathematics and Computer Engineering, Xihua University, Chengdu, Sichuan 610039, China; ^3^Department of Computing, Macquarie University, Sydney, NSW 2109, Australia

## Abstract

We develop a novel maximum neighborhood margin discriminant projection (MNMDP) technique for dimensionality reduction of high-dimensional data. It utilizes both the local information and class information to model the intraclass and interclass neighborhood scatters. By maximizing the margin between intraclass and interclass neighborhoods of all points, MNMDP cannot only detect the true intrinsic manifold structure of the data but also strengthen the pattern discrimination among different classes. To verify the classification performance of the proposed MNMDP, it is applied to the PolyU HRF and FKP databases, the AR face database, and the UCI Musk database, in comparison with the competing methods such as PCA and LDA. The experimental results demonstrate the effectiveness of our MNMDP in pattern classification.

## 1. Introduction

Dimensionality reduction (DR) plays an important role in many fields such as pattern classification, machine learning, and computer vision. Its purpose is to solve the “curse of dimensionality" [1] and map the high-dimensional points to a subspace that reveals the intrinsic structure of the original data. Among the DR techniques, principal component analysis (PCA) [2, 3], linear discriminant analysis (LDA) [3, 4], locality preserving projection (LPP) [5, 6], and their kernelized and tensorized variants [7–18] are the most representative and well-known algorithms. Although these methods have different suppositions, they can be put into a unified graph embedding framework with different constraints [19].

Generally, PCA aims to preserve the global geometric structure of data by maximizing the trace of the feature covariance matrix and produces compact representation of the original space in a low-dimensional space. However, it does not take into account the class label information. The goal of LDA is to find the global discriminant information for classification by maximizing the ratio between interclass and intraclass scatters. In contrast to PCA, LDA takes much consideration of the class information and strengthens the ability of pattern discrimination. Since both PCA and LDA only consider the global structure of data, they have little to do with the essential manifold of the data. As for them, it is difficult to discover the hidden submanifold that truly reflects the essential structure of the data.

In contrast to PCA and LDA, locality preserving projection (LPP) is introduced to detect the intrinsic geometry of the manifold structure of data [5, 6]. LPP is a classical liner graph embedding [19] derived from Laplacian Eigenmap [20]. It attempts to find an embedding that preserves the local neighborhood information and reflects the inherent submanifold structure. In recent years, many variants of LPP have been developed for dimensionality reduction [21–34]. These LPP-based DR algorithms can be mainly put into two categories: supervised and unsupervised ones. As for the supervised LPP-based methods, they generally employ class information of data in the process of subspace leaning, such as in [23–27, 29–32]. On the contrary, the unsupervised LPP-based ones do not consider class information [21, 22, 33, 34]. Among them, unsupervised discriminant projection (UDP) [21], as a simplified version of LPP [35], is a very popular method with the aim of resolving the “overlearning locality" existing in LPP. UDP seeks to find a projection by maximizing the nonlocal scatter and minimizing the local scatter simultaneously. As for LPP and UDP, since the local structure of data is modelled by the nearest neighbor graph, they may be not effective in the case of noisy data. Moreover, they are completely unsupervised in regard to the class label information and cannot perform well for classification. In addition, recently there are some new DR techniques that integrate sparse representation (SR) and subspace learning [36–41]. Based on the theory of sparse representation, these DR methods have more discriminating power for classification.

In this paper, we propose a novel dimensionality reduction method, named maximum neighborhood margin discriminant projection (MNMDP). It is based on the idea of LPP. However, unlike LPP, MNMDP is a supervised learning technique by fully utilizing the class label information for discovering the inherent manifold structure. The proposed MNMDP constructs a weighted *k*-nearest neighbor (*k*-NN) graph that models the data topology, and then the affinity weights of edges of the graph are built to fully capture local geometry of interclass and intraclass neighborhoods of each point in the phase of manifold learning. Although there exist many supervised variants of LPP, our MNMDP is very different from them in building adjacent similarities and the objective functions. In MNMDP, the affinity weight can be viewed as the combination of the local weight and the discriminating weight, the definition of which is different for intraclass and interclass neighbors of a given point. The local weight can well represent the local neighborhood structure, while the discriminating weight can further differentiate between different classes by using the class label information. Note that the definition of the affinity weight first appears in [23] and then is adopted to get good performance in face recognition in [26, 42]. After establishing the affinity weights of *k*-NN graph, we compute the intraclass neighborhood scatter and interclass neighborhood scatter, respectively. Then, a liner mapping for MNMDP is obtained by maximizing the margin between them. Hence, our MNMDP cannot only well preserve the intrinsic submanifold structures of data but also enhance the discrimination among different classes, so as to improve classification performance. The experimental results on four high-dimensional databases show that our proposed method performs well in pattern classification, compared to the competing methods: PCA, LDA, LPP, and UDP.

The rest of this paper is organized as follows.  Section 2 briefly reviews LPP. In Section 3, we introduce the proposed MNMDP method.  Section 4 represents in detail the classification performance of the competing methods by conducting comprehensive experiments.  Section 5 discusses the characteristics of the MNMDP. Finally, we conclude this paper in Section 6.

## 2. Locality Preserving Projection

For a general classification problem, denote by *X* = {*x*_*i*_ ∈ ℝ^*m*^}_*i*=1_^*N*^ the set of *N* *m*-dimensional samples, each of which has the class label *c*_*i*_ ∈ {1,2,…, *C*}, where *C* is the total number of classes. In general, the aim of dimensionality reduction is to transform the original *m*-dimensional space to a new *d*-dimensional subspace by a liner mapping; that is, *Y* = {*y*_*i*_ ∈ ℝ^*d*^}_*i*=1_^*N*^, where *d* ≪ *m*. The liner mapping for DR is always mathematically formulated as

(1) $$\begin{array}{c} y_{i}=\Phi^{T}x_{i}\;\text{for}\;\;i=1,\ldots ,N, \end{array}$$

where Φ ∈ ℝ^*m*×*d*^ is a transformation matrix and Φ = [*ϕ*_1_, *ϕ*_2_,…, *ϕ*_*d*_].

Nowadays, locality preserving projection (LPP) [5, 6] is a promising dimensionality reduction technique, which is a classical graph embedding [19]. Through a liner transformation projection, LPP can find an embedding that best discovers the intrinsical manifold structure of data. In order to do so, it first constructs a weighted affinity graph *𝒢* = (*𝒱*, *ℰ*), where *𝒱* is the set of all points of data and *ℰ* is the set of edges between any pairs of points. Note that the affinity graph *𝒢* is usually established by *k*-neighborhood; nodes *i* and *j* are connected by an edge if *x*_*i*_ is in *k* nearest neighbors of *x*_*j*_ or *x*_*j*_ is in *k* nearest neighbors of *x*_*i*_. Then, the weight *W*_*ij*_ of an edge between *x*_*i*_ and *x*_*j*_ is often defined as follows:

(2) $$\begin{array}{c} W_{ij}=\{\begin{array}{cc} \exp\left(-\frac{{||x_{i}-x_{j}||}^{2}}{\delta }\right), & x_{j}\in {𝒩}_{k}\left(x_{i}\right)\;\;\text{or}\;\;x_{i}\in {𝒩}_{k}\left(x_{j}\right)\\ 0, & \text{otherwise}, \end{array} \end{array}$$

where *𝒩*_*k*_(*x*_*i*_) or *𝒩*_*k*_(*x*_*j*_) denotes a set of the *k* nearest neighbors of the sample *x*_*i*_ or *x*_*j*_ and the parameter *δ* is a positive constant.

To obtain a liner graph embedding, the objective function of LPP to be minimized is as follows:

(3) $$\begin{array}{c} \min\underset{ij}{\sum }{||y_{i}-y_{j}||}^{2}W_{ij}. \end{array}$$

The criterion above with *W*_*ij*_ can give a penalty for mapping neighboring points *x*_*i*_ and *x*_*j*_ far apart in an embedded subspace. By simple algebra, the objective criterion for LPP under the appropriate constraint can be rewritten as

(4) argmin⁡Φ tr⁡(ΦTXLXTΦ)s.t.     ΦTXDXTΦ=I,

where *D* is a diagonal matrix and its entries are column (or row, since *W* is symmetric) sum of *W*; that is, *D*_*ii*_ = ∑_*j*_ *W*_*ij*_ and *L* = *D* − *W* are the Laplacian matrix. In (4), tr⁡(·) and *I* denote the trace of the matrix and the identity matrix, respectively.

Finally, the transformation matrix Φ to minimize (4) can be achieved by solving the generalized eigenvalue problem

(5) $$\begin{array}{c} XLX^{T}\Phi =\lambda XDX^{T}\Phi , \end{array}$$

where Φ only contains *d* eigenvectors corresponding to the *d* smallest eigenvalues, that is, *λ*_1_ ≤ *λ*_2_ ≤ ⋯≤*λ*_*d*_.

## 3. Maximum Neighborhood Margin Discriminant Projection

In pattern recognition, the class label information of data plays an important role for classification. Moreover, the local structures of the training samples are also very useful for it. Inspired by the two facts, a novel dimensionality reduction method, called maximum neighborhood margin discriminant projection (MNMDP), is introduced in this section. In the proposed MNMDP, both the class information and local geometry of data are fully taken into account for classification simultaneously. The major focus of MNMDP is to seek a liner graph embedding that not only detects the underlying submanifold structure of data from the same class but also enhances the discrimination among submanifolds from different classes in the process of the learning. To obtain this graph embedding of given data, it first constructs the *k*-nearest neighbor graph of all points that can be retained in the projected subspace, and then assigns completely distinct weights for the interclass and intraclass neighbors of a point by fully considering the class information, and at last finds a liner mapping by maximizing the margin between the interclass and intraclass neighbors of all points, so as to improve the classification performance in the new subspace.

### 3.1. Intraclass Neighborhood Scatter

Given a data set *X* = {*x*_*i*_ ∈ ℝ^*m*^}_*i*=1_^*N*^, MNMDP first begins to build an adjacent graph *𝒢* by *k*-neighborhood for all points. For a data point *x*_*i*_, let *𝒩*_*k*_(*x*_*i*_) be the set of *k*-nearest neighbors of it. In the meantime, let *𝒩*_*k*_^+^(*x*_*i*_) denote the intraclass neighbors in the *k*-neighborhood *𝒩*_*k*_(*x*_*i*_) (i.e., neighbors from the same class as *x*_*i*_) and *𝒩*_*k*_^−^(*x*_*i*_) the interclass neighbors of *x*_*i*_ in *𝒩*_*k*_(*x*_*i*_) (i.e., neighbors from different classes). In order to represent the neighborhood relationship of each data point, we need to build an affinity matrix for intraclass neighborhood and interclass neighborhood, respectively. Then, the intraclass and interclass neighborhood scatters are accordingly computed to preserve the local neighborhood structures of the data. Subsequently, by keeping the margin between the intraclass and interclass neighborhood maximum with two scatters, MNMDP can possess important pattern discrimination for classification in the projected subspace.

The affinity weights for intraclass neighborhoods of all points are defined as follows:

(6) $$\begin{array}{c} W_{ij}^{+}=\{\begin{array}{c} \exp\left(-\frac{{||x_{i}-x_{j}||}^{2}}{\delta }\right)\left(1+\exp\left(-\frac{{||x_{i}-x_{j}||}^{2}}{\delta }\right)\right),\\ x_{j}\in {𝒩}_{k}^{+}\left(x_{i}\right)\;\;\text{or}\;\;x_{i}\in {𝒩}_{k}^{+}\left(x_{j}\right),\\ 0,\;\;\;\;\;\;\text{otherwise}, \end{array} \end{array}$$

where the parameter *δ* is a positive regulator. According to (6), the affinity weight integrates the local weight, that is, exp⁡(−||*x*_*i*_−*x*_*j*_||^2^/*δ*), which can preserve the local intraclass neighborhood structure and the intraclass discriminating weight, that is, 1 + exp⁡(−||*x*_*i*_−*x*_*j*_||^2^/*δ*), which can represent the class information of the same classes. From (6), the affinity weights of the intraclass points are larger than those in LPP. This fact is very advantageous to classification.

From the viewpoint of pattern recognition, it is quite favorable for presuming that the different samples have different contributions to classification. Generally speaking, the fact that the samples with greater contributions have the more significance for classification is naturally related to their neighborhood location in the feature space. Here, we take into account a local scaling regulator of data to dynamically adjust adjacent weights between pairs of neighbors, so as to reasonably reflect the classification contribution of each sample. According to the *k*-neighborhood of one sample *x*_*i*_, the parameter *δ* as local scaling regulator in (6) is set to be as follows:

(7) $$\begin{array}{c} \delta =\frac{1}{k^{2}}\overset{k}{\underset{j=1}{\sum }}{||x_{i}-x_{j}||}^{2}. \end{array}$$

This can be a good reasonable way to estimate the value of *δ*, and the affinity weights *W*_*ij*_^+^ between nodes *i* and *j* are allowed to self-tune in terms of the *k*-neighborhood with *δ*.

To still retain the intraclass neighborhood relations through a liner mapping, that is, *y*_*i*_ = Φ^*T*^ *x*_*i*_, the intraclass neighborhood scatter along a projection *ϕ* is defined as

(8) $$\begin{array}{c} J^{+}\left(\phi \right)=\frac{1}{2}\underset{ij}{\sum }{||y_{i}-y_{j}||}^{2}W_{ij}^{+}. \end{array}$$

It follows from (8) that

(9) J+(ϕ)=12∑ij||ϕTxi−ϕTxj||2Wij+=ϕT[12∑ij(xi−xj)(xi−xj)TWij+]ϕ=ϕTS+ϕ,

where *S* ^+^ is called the intraclass neighborhood scatter matrix:

(10) $$\begin{array}{c} S^{+}=\frac{1}{2}\underset{ij}{\sum }\left(x_{i}-x_{j}\right){\left(x_{i}-x_{j}\right)}^{T}W_{ij}^{+}. \end{array}$$

To gain more insight into (10) in terms of the affinity matrix *W* ^+^ from (6), *S* ^+^ is rewritten as

(11) S+=12(∑ijWij+xixiT+∑ijWij+xjxjT−2∑ijWij+xixjT)=∑iDii+xixiT−∑ijWij+xixjT=XD+XT−XW+XT=XL+XT,

where *D* ^+^ is a diagonal matrix, its elements are column sum of *W* ^+^, that is, *D*_*ii*_^+^ = ∑_*j*_ *W*_*ij*_^+^, and *L* ^+^ = *D* ^+^ − *W* ^+^. Note that *L* ^+^, *D* ^+^, and *S* ^+^ are symmetric matrixes.

In order to well preserve the intraclass neighborhood and keep the intraclass neighborhood scatter compact in the embedded subspace, the optimal projections can be obtained by minimizing the intraclass neighborhood scatter:

(12) $$\begin{array}{c} \mathrm{Min}J^{+}\left(\phi \right)=\phi^{T}S^{+}\phi . \end{array}$$

### 3.2. Interclass Neighborhood Scatter

In contrast to intraclass neighborhood scatter, the affinity weights for interclass neighborhoods of all points are defined as follows:

(13) $$\begin{array}{c} W_{ij}^{-}=\{\begin{array}{c} \exp\left(-\frac{{||x_{i}-x_{j}||}^{2}}{\delta }\right)\left(1-\exp\left(-\frac{{||x_{i}-x_{j}||}^{2}}{\delta }\right)\right),\\ x_{j}\in {𝒩}_{k}^{-}\left(x_{i}\right)\;\;\text{or}\;\;x_{i}\in {𝒩}_{k}^{-}\left(x_{j}\right),\\ 0,\;\;\;\;\;\;\text{otherwise}, \end{array} \end{array}$$

where the parameter *δ* is a positive regulator, the same as (7). In (13), the affinity weight can simultaneously reflect the local interclass neighborhood structure by the local weight exp⁡(−||*x*_*i*_−*x*_*j*_||^2^/*δ*) and the class information of the different classes by interclass discriminating weight 1 − exp⁡(−||*x*_*i*_−*x*_*j*_||^2^/*δ*). From (13), the fact that the affinity weights of the interclass points are less than those in LPP is also very helpful for classification.

Then, the interclass neighborhood scatter along a projection *ϕ* is defined as

(14) $$\begin{array}{c} J^{-}\left(\phi \right)=\frac{1}{2}\underset{ij}{\sum }{||y_{i}-y_{j}||}^{2}W_{ij}^{-}. \end{array}$$

It follows from (14) that

(15) J−(ϕ)=12∑ij||ϕTxi−ϕTxj||2Wij−=ϕTS−ϕ,

where *S* ^−^ is called the interclass neighborhood scatter matrix:

(16) $$\begin{array}{c} S^{-}=\frac{1}{2}\underset{ij}{\sum }\left(x_{i}-x_{j}\right){\left(x_{i}-x_{j}\right)}^{T}W_{ij}^{-}. \end{array}$$

By the same algebra as *S* ^+^, *S* ^−^ in (16) is rewritten as follows:

(17) $$\begin{array}{c} S^{-}=XL^{-}X^{T}, \end{array}$$

where *D* ^−^ is a diagonal matrix, its elements are column sum of *W* ^−^, that is, *D*_*ii*_^−^ = ∑_*j*_ *W*_*ij*_^−^, and *L* ^−^ = *D* ^−^ − *W* ^−^. Note that *L* ^−^, *D* ^−^, and *S* ^−^ are symmetric matrixes.

To gain more discrimination between different classes through a liner mapping, the interclass neighborhood scatter in the projected subspace should be kept more separable by maximizing the following criterion:

(18) $$\begin{array}{c} \mathrm{Max}J^{-}\left(\phi \right)=\phi^{T}S^{-}\phi . \end{array}$$

### 3.3. Optimal Liner Embedding

Combining (12) and (18) with the orthonormal constraint (i.e., Φ^*T*^Φ = *I*), we get the following objective function:

(19)  Min⁡ J+(Φ)=tr⁡(ΦTS+Φ) Max⁡ J−(Φ)=tr⁡(ΦTS−Φ) s.t.  ΦTΦ=I,

where Φ = [*ϕ*_1_, *ϕ*_2_,…, *ϕ*_*d*_] and *ϕ*_*i*_ is an orthogonal vector. Based on the idea of the maximum margin criterion [43], (19) can be reformulated as follows:

(20) argmax⁡Φ tr⁡(ΦT(S−−S+)Φ)s.t.    ΦTΦ=I,

or

(21)  max⁡ ∑i=1dϕiT(S−−S+)ϕi s.t.  ϕiTϕi=1, ϕiTϕj=0 (i≠j).

According to (20) or (21), we can find two aspects that are favorable for classification. On one hand, the optimal projections obtained are such that the intraclass samples are attracted being more compact (minimizing the intraclass neighborhood scatter), while the interclass samples are simultaneously pulled being more separable (maximizing the interclass neighborhood scatter). Of course, it can keep the margin between intraclass and interclass neighborhood maximum in a new subspace, so as to clearly enhance pattern discrimination. On the other hand, the graph embedding obtained with orthogonal projections can have both more locality preserving power and more discriminating power [33].

To maximize the above objective function, we can use the Lagrangian multiplier method to first build the following function:

(22) $$\begin{array}{c} L\left(\phi_{i},\lambda_{i}\right)=\overset{d}{\underset{i=1}{\sum }}\left(\phi_{i}^{T}\left(S^{-}-S^{+}\right)\phi_{i}-\lambda_{i}\left(\phi_{i}^{T}\phi_{i}-1\right)\right), \end{array}$$

where *λ*_*i*_  (*i* = 1,…, *d*) is a Lagrange multiplier. Then, the optimization is carried out by the partial derivative of *L*(*ϕ*_*i*_, *λ*_*i*_) with respect to *ϕ*_*i*_:

(23) $$\begin{array}{c} \frac{\partial L\left(\phi_{i},\lambda_{i}\right)}{\partial \phi_{i}}=\left(S^{-}-S^{+}\right)\phi_{i}-\lambda_{i}\phi_{i}. \end{array}$$

Let (23) be zero; we yield

(24) $$\begin{array}{c} \left(S^{-}-S^{+}\right)\phi_{i}=\lambda_{i}\phi_{i}. \end{array}$$

Thus, the optimal matrix Φ that maximizes the objective criterion in (20) can be achieved by solving the generalized eigenvalue problem

(25) $$\begin{array}{c} \left(S^{-}-S^{+}\right)\Phi =\lambda \Phi , \end{array}$$

where Φ only contains *d* eigenvectors corresponding to the *d* largest positive eigenvalues, that is, *λ*_1_ ≥ *λ*_2_ ≥ ⋯≥*λ*_*d*_ ≥ 0. Note that, since the symmetric matrix (*S* ^−^ − *S* ^+^) is not positive semidefinite, the eigenvalues of (*S* ^−^ − *S* ^+^) may be positive, negative, or zero. To maximize (20), we only need to select the *d* largest positive eigenvalues.

### 3.4. The MNMDP Algorithm

Based on the above description, the algorithmic procedure of the proposed MNMDP is summarized in Algorithm 1.

## 4. Experimental Results

In this section, we evaluate the classification performance of the proposed MNMDP, in comparison with the state-of-the-art DR methods: PCA, LDA, LPP, and UDP. The experiments are conducted on four databases including HRF, FKP, AR, and Musk databases. In order to measure the recognition performance, *l* images per class on each database are randomly selected as training samples, while the remaining images per class are used for testing. To ensure that the performance is not biased from certain random realization of training/testing set, 10 independent runs for a given *l* are performed. Since training and testing samples are chosen randomly online, those 10 runs give us 10 different training and testing sets for performance evaluation. Note that both training and testing sets on each run have no overlap between them. The average recognition rates across these 10 runs with 95% confidence are regarded as the final classification results. In our experiments, to overcome the small sample size problem encountered in LDA, LPP, and UDP, PCA preprocessing is employed to solve the singular matrix by keeping nearly 98% image energy. If the singularities still exist, pseudoinverse is adopted. In order to ensure a fair comparisons, the regulator *δ* in LPP is set in terms of (7). The *k*-neighborhood parameter denoted by *Wk* for building the weight matrix in LPP and UDP is selected as *Wk* = *l* − 1 [21]. The nearest neighbor (NN) classifier with Euclidean distance is used for classification in the experiments.

### 4.1. Experiments on HRF

**Figure 1:**
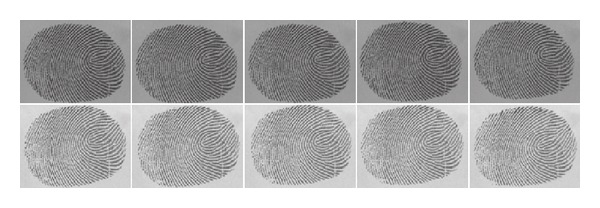
Ten sample images of one subject in the HRF database.

The PolyU HRF (High-Resolution-Fingerprint) database [44] was collected in two separate sessions. Here, we use the DBII of HRF. The database contains 148 fingers, each of which has five sample images per session. Each image is taken with 1200 dpi and the size per image is 640 × 480 pixels, with 256 grey levels per pixel. For computational efficiency, each image is resized to 32 × 32 pixels in our experiments. As an example, Figure 1 shows ten images of one finger in the HRF database. We form the training set by a random subset of *l* images per class and use the rest as a testing set. In the following experiments, the numbers of training sample images per class are chosen as *l* = 5, 6,7, 8.

**Figure 2:**
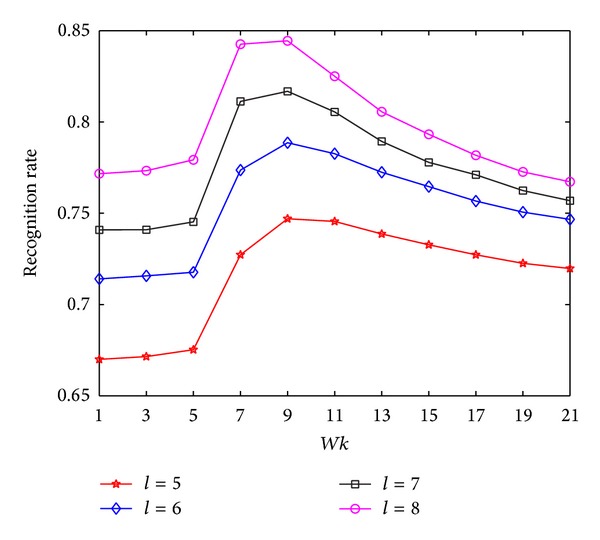
The average recognition rates of MNMDP versus *Wk* on HRF.

In the experiments on HRF, we first explore the performance of MNMDP with varying *k*-neighborhood parameter *Wk* in terms of recognition rates. The value of *Wk* is set from 1 to 21 in Step 2. The maximal average classification results via *Wk* for each *l* are plotted in Figure 2. It can be obviously observed that the proposed MNMDP with more training samples has better classification. As can be seen in Figure 2, the performance of MNMDP for each *l* first increases slowly when *Wk* changes from 1 to 5, and then increases rapidly when *Wk* changes from 5 to 9, and finally drops with increase of *Wk*. The possible reason for this experimental phenomenon is that the affinity graph is unable to capture effectively the geometry of data when *Wk* is small and the more geometrical information of data can be preserved as *Wk* increases. However, when *Wk* is beyond the reasonable value, the *k*-neighborhood for a given point may include more interclass points [45], and this can degrade the ability of pattern discrimination. Consequently, the experimental results reveal that *k*-neighborhood parameter *Wk* in MNMDP plays an important role for preservation of geometrical and discriminant information of data that is available for classification and its suitable value can be easily selected to achieve good performance.

**Figure 3:**
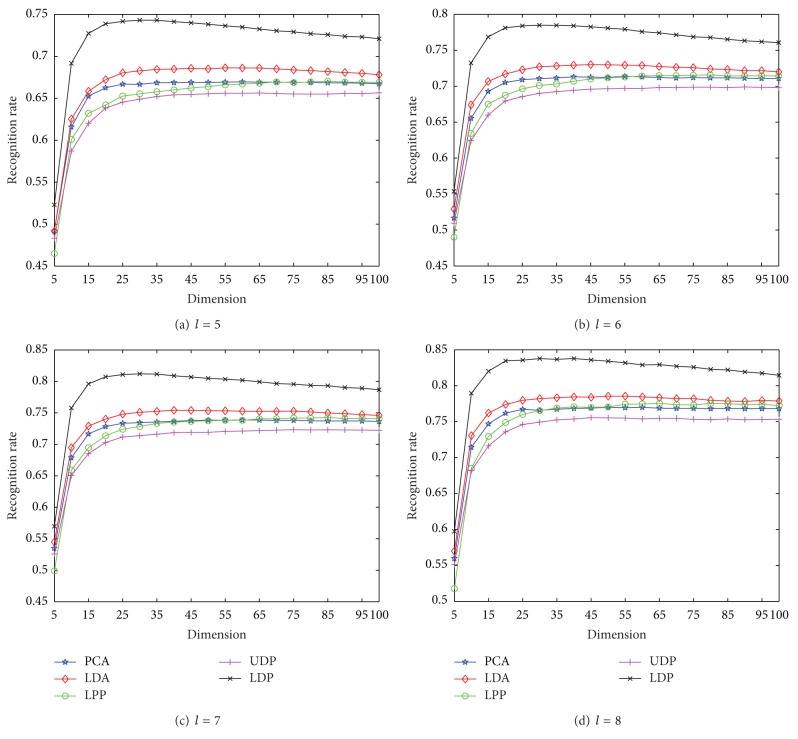
The average recognition rates versus dimension on HRF.

Furthermore, the experimental comparisons of competing methods are studied by varying the reduced dimensionality on HRF. We experiment with the dimension of the reduced space from 5 to 100 in Step 5. Based on the results shown in Figure 2 the best *k*-neighborhood parameters for MNMDP are set as *Wk* = 9 for *l* = 5,6, 7,8, respectively. For each *l* on HRF, the comparative average recognition performance of each method is given in Figure 3. As shown in Figure 3, the classification performance of each method ascends quickly until the dimensionality is about 30 and then keeps almost stable or decreases slowly with increase of dimensionality. It is clear that the proposed MNMDP consistently outperforms the other methods at any value of dimensionality, making the superiority of the MNMDP evident. Observe again that LDA obtains the better performance than PCA, LPP, and UDP, PCA is preferable to LPP at small values of dimension while they get the quite similar performance at large values of dimension, and UDP is the weakest among them. This fact that the performance of MNMDP and LDA is better than that of PCA, LPP, and UDP may be because our MNMDP and LDA are supervised learning methods using class information. Thereby, the experimental results in Figure 3 demonstrate that MNMDP is more robust over a large range of dimensionality with satisfactory performance.

The best performance of the competing methods by means of the highest average recognition rates with the corresponding standard deviations (stds) and values of dimension in the parentheses is also given in Table 1 for each *l* on HRF. Note that the best recognition rates for each *l* among the methods are marked in bold face. We can obviously see that the performance of each method increases with increase of the training samples. As expected, the proposed MNMDP achieves the best performance and the corresponding dimensionality is the smallest among the competing methods. It can also be observed from Table 1 that LDA is better than PCA, LPP, and UDP and LPP is superior to PCA. In addition, UDP is better than LPP and PCA when *l* = 5 and less than them when *l* = 6,7, 8. As a consequence, the promising performance of our MNMDP is confirmed on HRF.

### 4.2. Experiments on FKP

**Figure 4:**
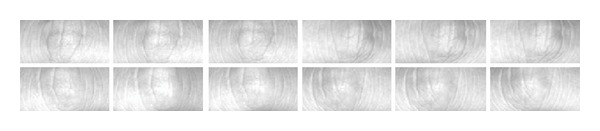
Twelve sample images of one subject in the FKP database.

The PolyU FKP (Finger-Knuckle-Print) database [46] contains 165 subjects, each of which has 48 samples that were taken in two separate sessions. Each session per subject has 6 images for each of the left index finger, the left middle finger, the right index finger, and the right middle finger. Here, we use FKP ROI database obtained by ROI extraction algorithm in [47] and the size of each image is 220 × 110 pixels, with 256 grey levels per pixel. To save computation time, we use a subset of the FKP ROI database in the experiment. The data set we selected contains the 100 subjects and 12 images for each individual. Note that the first 3 sample images per finger are selected. In our experiments, each image is resized to 32 × 32 pixels for computational efficiency. As an example, Figure 4 shows twelve images of one subject in the FKP database. We form the training set by a random subset of *l* images per class and use the rest as a testing set. In the following experiments on FKP, the numbers of training sample images per class are chosen as *l* = 5, 7,9, 11.

**Figure 5:**
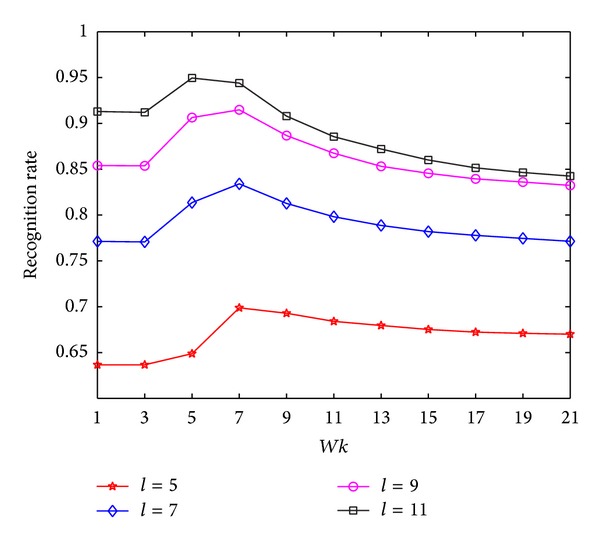
The average recognition rates of MNMDP versus *Wk* on FKP.

In the experiments on FKP, we first investigate the recognition performance of MNMDP on FKP by varying the *k*-neighborhood parameter *Wk*. The value of *Wk* is varied from 1 to 21 in Step 2. The maximal average recognition rate at each value of *Wk* for each *l* is illustrated in Figure 5. It is obvious that the classification performance of MNMDP increases when the number of training samples increases. As can be noted in Figure 5, the performance of MNMDP nearly increases when *Wk* changes from 1 to 7 at first and then decreases when *Wk* becomes large. From Figure 5, it can be concluded that the appropriate value of *Wk* in MNMDP is significant for capturing the geometrical structure and pattern discrimination of data on FKP, the same as on HRF, and it can be easily selected to obtain good performance.

**Figure 6:**
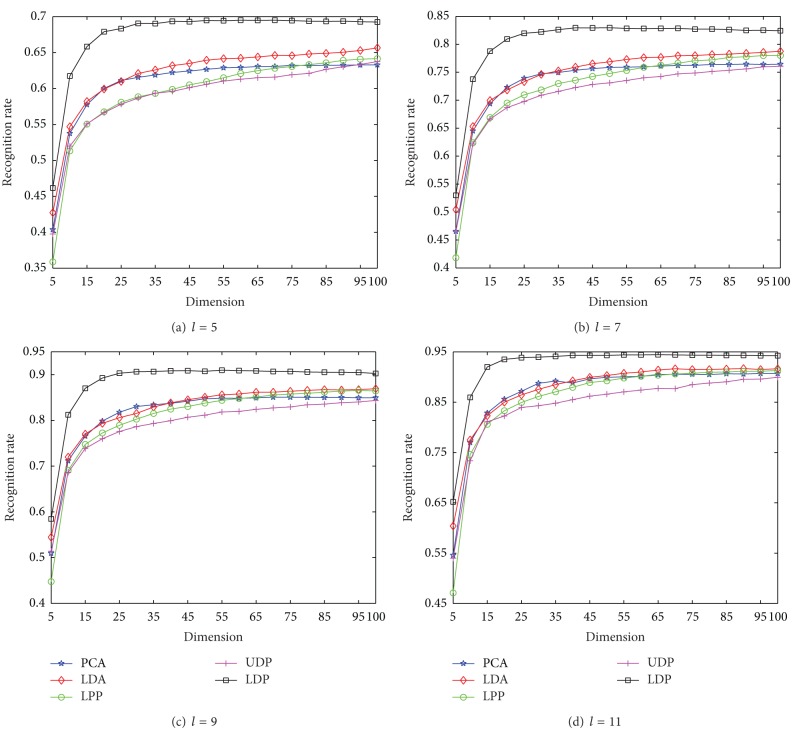
The average recognition rates versus dimension on FKP.

To further verify the performance of MNMDP, the comparative classification results of the competing methods on FKP are reported in Figure 6 with varying the reduced dimensionality from 5 to 100 in Step 5. According to the experimental results in Figure 5, the best *k*-neighborhood parameters for MNMDP are set as *Wk* = 7 for *l* = 5,7, 9 and *Wk* = 5 for 11, respectively. We can see that the classification performance of each method almost ascends monotonically with increasing the dimensionality, at first increases quickly, and finally increases very slowly or even keeps stable. It is noticeable that the proposed MNMDP outperforms the other methods significantly across all values of dimensionality for each *l*, and UDP almost obtains the worst performance among them. We can also observe that the performance of LDA is always superior to that of LPP and UDP over a large range of dimensionality, and PCA performs better than LPP when the dimension is small whereas it does worse when the dimension is large. Based on the experimental comparisons in Figure 6, the conclusion we have drawn is that our method consistently obtains better classification performance, irrespective of the variation in dimensions.

For each *l* on FKP, the experimental comparisons of the competing methods in terms of the maximal average recognition rates with the corresponding standard deviations (stds) and values of dimension in the parentheses are also tabulated in Table 2. It should be noted that the best performance for each *l* among the methods is indicated in bold face. It can be seen from Table 2 that the proposed MNMDP is very superior to the other methods and its optimal reduced dimensionality is far smaller than that of them. Moreover, the performance of each method increases as the number of the training samples increases. We can also observe that the best performance of LDA is better than that of PCA, LPP, and UDP and UDP and LPP are preferable to PCA. Through the comparative study of the best performance of the competing methods, we can conclude that the MNMDP has more discriminating power to achieve the satisfactory classification.

### 4.3. Experiments on AR

**Figure 7:**
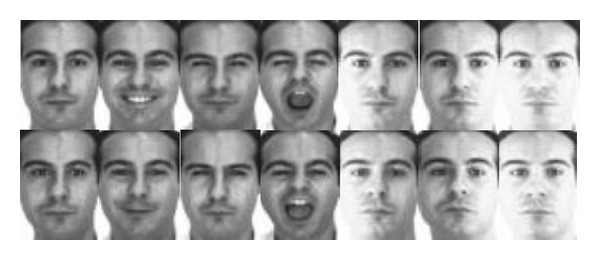
Fourteen sample images of one subject in the AR database.

The AR face database [48] contains over 4,000 color images corresponding to 126 people's faces (70 men and 56 women). The image samples of each person were taken in two sessions, separated by two weeks time. Here, we select a subset of AR including 50 men and 50 women, and each person has 14 image samples, separately collected in two sessions with neutral expression, smile, anger, and scream, left light on, right light on, and all side lights on. Each image is manually cropped and then normalized to 32 × 32 pixels, with 256 grey levels per pixel. As an example, Figure 7 shows the images of one person in the AR database. We form the training set by a random subset of *l* images per class and use the rest as a testing set. In the following experiments on AR, the numbers of training sample images per class are chosen as *l* = 7, 9,11,13.

**Figure 8:**
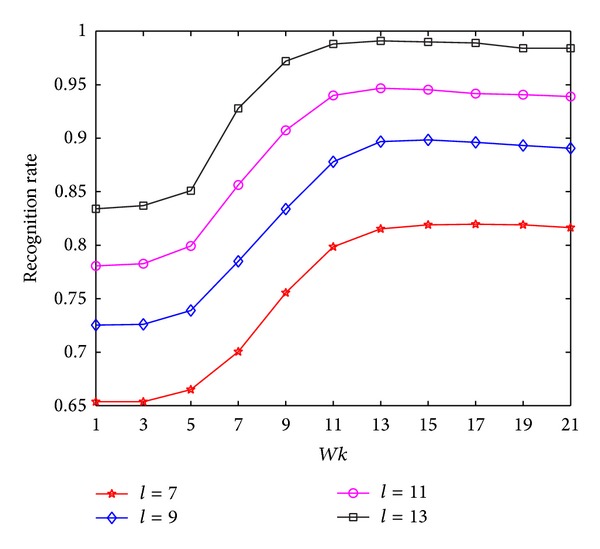
The average recognition rates of MNMDP versus *Wk* on AR.

In the experiments on AR, the classification performance of the proposed MNMDP versus the *k*-neighborhood parameter *Wk* is first carried out for each *l*, shown in Figure 8. Notice that the values of *Wk* are varied from 1 to 21 in Step 2. It is obvious that the performance of MNMDP increases with the increase of the training samples. As can be noted in Figure 8, the recognition rates of MNMDP ascend quickly at first with increase of *Wk* and then almost keep stable when *Wk* becomes large. Thus, we can conclude that the appropriate value of *Wk* plays an important role in MNMDP for preserving the geometry of data and enhancing the power of pattern discrimination, and it can be easily set to obtain good performance.

**Figure 9:**
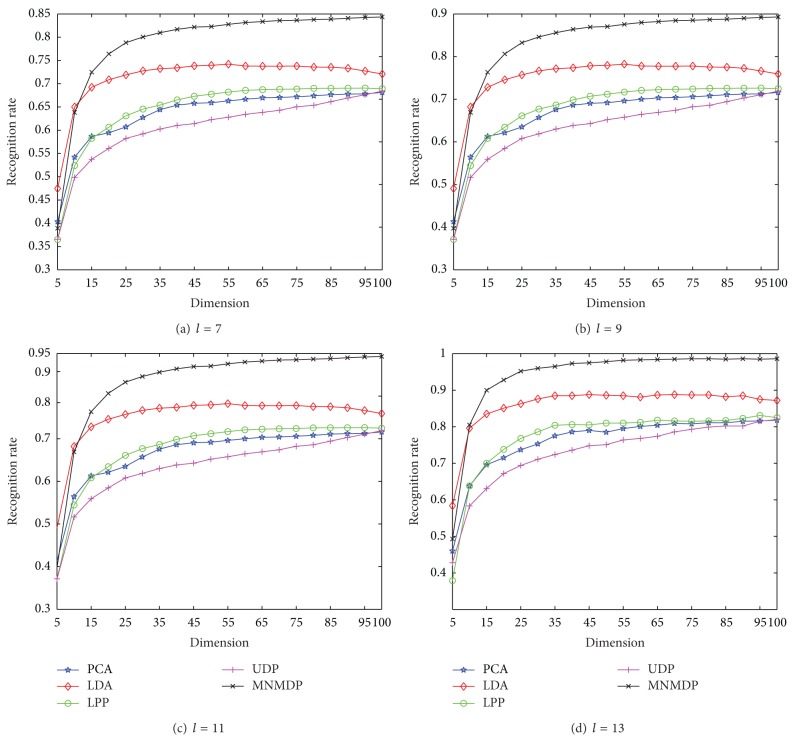
The average recognition rates versus dimension on AR.

Moreover, the classification performance of the proposed MNMDP is further evaluated on AR by varying the reduced dimensionality, in comparison with the competing methods. The dimensionality varies from 5 to 100 with an interval of 5. Note that from the results in Figure 8, the best *k*-neighborhood parameters for MNMDP are set as *Wk* = 17 for *l* = 7, *Wk* = 15 for *l* = 9, and *Wk* = 13 for *l* = 11,13, respectively. The performance of each method in terms of average recognition rates is illustrated in Figure 9. It can be seen that the performance of each method for each *l* first increases rapidly when *Wk* becomes large and then approximately tends to be stable. Compared to PCA, LDA, LPP, and UDP, the proposed MNMDP method almost has the best performance by varying the dimensionality, especially at the large values of dimensionality. In the meantime, LDA is superior to PCA, LPP, and UDP with increasing the dimensionality. In addition, in most cases LPP is better than PCA and UDP, and PCA is better than UDP. From the comparative performance in Figure 9, we can conclude that our method always has better classification results over a large range of the dimensionality.

The maximal average recognition rates of each competing method on AR for each *l* with the corresponding standard deviations (stds) and values of dimension in the parentheses are also reported in Table 3. It is to be noted that the best performance among them is described in bold face. We can see that the performance of each method is improved by increasing the number of the training samples. As Table 3 displays, MNMDP has the best performance among the methods for each *l*. It can also be observed that the best classification performance of LDA is better than that of LPP, PCA, and UDP. Consequently, the experimental results in Figure 9 and Table 3 on AR face database certainly demonstrate the good performance of the proposed MNMDP.

### 4.4. Experiments on Musk

The Musk (version1) database [49] is one of the two-class classification tasks that predicts whether new molecules will be musks or nonmusks. It totally contains 476 samples, each of which has 166 attributes that depend on the exact shape or conformation of these molecules. In the experiments, we set the number of training samples per class as *l* = 50,80,110,140, respectively, and the remaining samples are used to test the competing methods.

**Figure 10:**
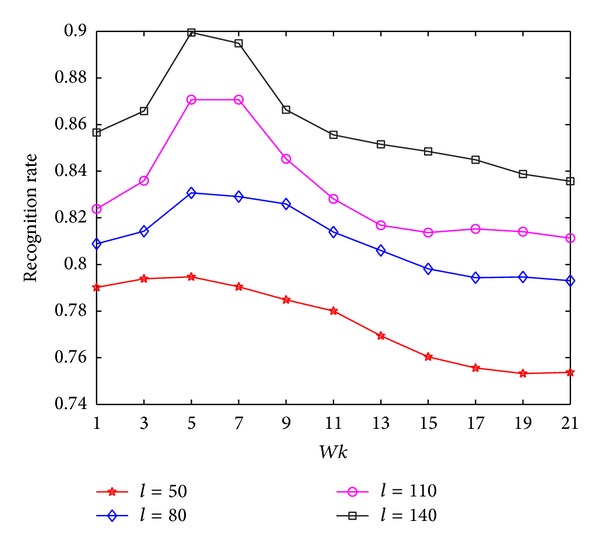
The average recognition rates of MNMDP versus *Wk* on Musk.

In the experiments on Musk, we first investigate the classification performance of the proposed MNMDP versus the *k*-neighborhood parameter *Wk* for each *l*. The experimental results are shown in Figure 10. The values of *Wk* are presented from 1 to 21 in Step 2. As can be seen in Figure 10, the recognition rates of MNMDP increase from 1 to 5 and then drop when the values of *Wk* increase. Hence, the classification results have revealed that the *k*-neighborhood parameter *Wk* is very important for MNMDP to preserve the geometrical structures of data and to strengthen pattern discrimination, and the appropriate value of *Wk* for good performance can be easily determined.

**Figure 11:**
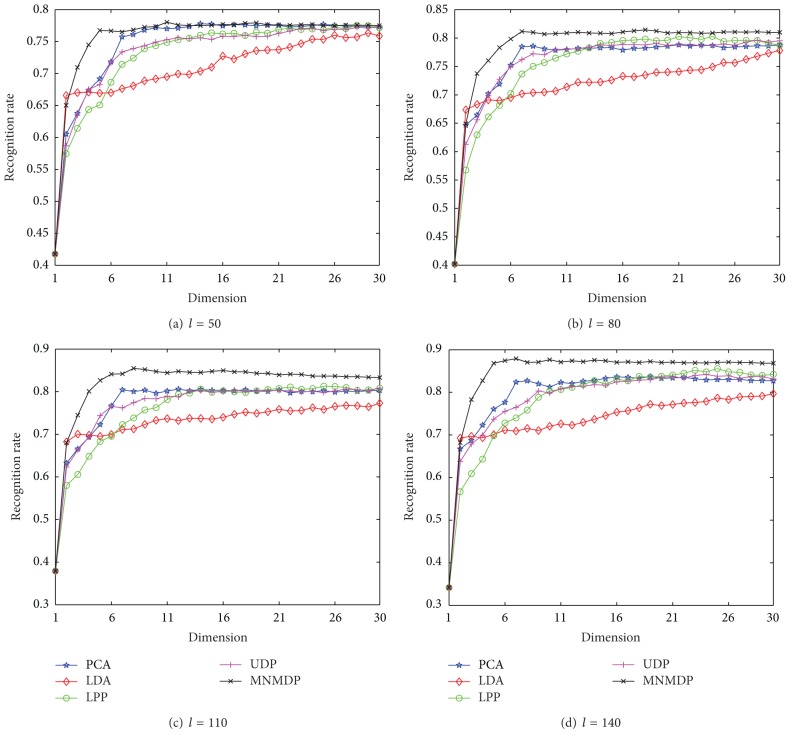
The average recognition rates versus dimension on Musk.

To further verify the classification performance of our MNMDP on Musk, it is compared to the competing methods by varying the reduced dimensionality. The dimensionality increases from 1 to 30 in Step 1. It should be noted that from the results in Figure 10, the best *k*-neighborhood parameters for MNMDP are determined as *Wk* = 5 for *l* = 50,80, 110,140, respectively.  Figure 11 shows the performance of each method in terms of average recognition rates. It can be found that the performance of each method for each *l* first ascends at small values of *Wk* and then approximately tends to be stable or increases slowly when *Wk* becomes large. As shown in Figure 11, the proposed MNMDP method almost has the best performance by varying the dimensionality among all the methods. It can also be observed that PCA, LPP, and UDP get the similar performance when *Wk* is about larger than 10, and LDA obtains the worse performance when *Wk* varies from 6 to 30. Therefore, the classification results in Figure 11 indicate that our method is always better than PCA, LDA, LPP, and UDP with the change of dimensionality.

The comparative experiments on Musk for each *l* in terms of the maximal average recognition rates with the corresponding standard deviations (stds) and values of dimension in the parentheses are finally shown in Table 4. Note that the best performance for each *l* among all the methods is represented in bold face. It is clear that the classification performance of the proposed MNMDP is better than PCA, LDA, LPP, and UDP. In the meantime, the optimal reduced dimensionality of our MNMDP for each *l* is smaller than that of them. Therefore, we can conclude that the proposed MNMDP does well in dimensionality reduction with good classification.

In summary, the proposed MNMDP almost yields the best classification performance in all the experiments, compared to PCA, LDA, LPP, and UDP. It implies that both pattern discrimination and geometrical information of the data are very important for classification, and MNMDP fully captures them in the learning processing.

## 5. Discussions

In this section, some characteristics of the proposed MNMDP that are available for classification are discussed. We first analyze the affinity weight for intraclass and interclass neighborhoods, then discuss the MNMDP from the viewpoint of distance metric learning [50, 51], and finally explore the eigenvalues of the generalized eigenvalue problem in MNMDP.

According to (6) and (13), the affinity weight can be thought of as integration of the local weight exp⁡⁡(−||*x*_*i*_−*x*_*j*_||^2^/*δ*) and the discriminating weight that is divided into two categories: intraclass one for intraclass neighbors 1 + exp⁡⁡(−||*x*_*i*_−*x*_*j*_||^2^/*δ*) and interclass one for interclass neighbors 1 − exp⁡⁡(−||*x*_*i*_−*x*_*j*_||^2^/*δ*). It means that the affinity weight cannot only preserve local structures of data but also distinguish between different classes. Through the analysis of the affinity weight in MNMDP, its three properties can be summarized as follows [23].

**Property 1**

For a given point, the affinity weight gives more similarity to the intraclass neighbors than the interclass ones, when the Euclidean distances are equivalent. This is favorable for classification.

**Property 2**

The affinity weight can retain intraclass and interclass similarity in certain ranges no matter how strong the noise is, since 1 ≤ 1 + exp⁡⁡(−||*x*_*i*_−*x*_*j*_||^2^/*δ*) ≤ 2 and 0 ≤ 1 − exp⁡(−||*x*_*i*_−*x*_*j*_||^2^/*δ*) ≤ 1. This can largely preserve local geometric structures of data for different classes.

**Property 3**

The affinity weight can strengthen the ability of margin augmentation and noise suppression. This is explained by two aspects. One is that the close points from different classes could have smaller values of similarity and the margin between different classes can be augmented, because 1 − exp⁡(−||*x*_*i*_−*x*_*j*_||^2^/*δ*) tends towards 0 with a decrease of the Euclidean distance. The other is that the affinity weight can inhibit the noise in some degree; that is, the more distant points from the same class could be less similar to each other, because exp⁡(−||*x*_*i*_−*x*_*j*_||^2^/*δ*) tends towards 0 with an increase of the Euclidean distance.

The above good properties make a good supervised construction of the affinity graph, and they are well maintained in the process of MNMDP subspace learning. They can overcome the issue that the interclass neighbors might have the same representation as the intraclass neighbors in the reduced subspace for the given point in LPP [52]. Based on them, MNMDP achieves a good projection that carries not only local geometric structures but also discriminant information. Thus, the properties of the affinity weight make the MNMDP robust and effective for the classification tasks.

In regard to classification, the MNMDP can be viewed as one method of distance metric learning [50, 51]. Once the transformation matrix Φ is obtained by MNMDP, the squared distance between *x*_*i*_ and *x*_*j*_ in the embedded subspace can be formulated as follows:

(26) d(yi,yj)=||ΦTxi−ΦTxj||2=(xi−xj)TΦΦT(xi−xj)=(xi−xj)TM(xi−xj)=||xi−xj||M2,

where *M* = ΦΦ^*T*^. Thus, finding a liner transformation Φ in MNMDP is equivalent to learning the distance metric *M* implicitly. This is very important in pattern classification. Moreover, making the projection orthogonal (i.e., Φ^*T*^Φ = *I*) will tend to preserve distances between any points *x*_*i*_ and *x*_*j*_ in the projected subspace [53]. Due to linearity, the overall geometry of the data will also tend to be preserved. It should be noted that there are many distance metric learning methods that can be used for dimensionality reduction, such as neighborhood components analysis (NCA) [54] and large margin nearest neighbor classification (LMNNC) [55]. Since NCA and LMNNC closely connect to the *k*-NN classifier and their objective functions and optimization solutions are very different from MNMDP, more comparative discussions between them are beyond the scope of this paper here.

Given that data points are projected along an eigenvector *ϕ*_*i*_ corresponding to eigenvalue *λ*_*i*_, it follows from (24) with the orthogonal constraint *ϕ*_*i*_^*T*^ *ϕ*_*i*_ = 1 that

(27) J(ϕi)=J−(ϕi)−J+(ϕi)=ϕiT(S−−S+)ϕi=ϕiTλiϕi=λi.

It is clear that *J*(*ϕ*_*i*_) characterizes the margin between interclass and intraclass neighborhoods for all the points along the projection *ϕ*_*i*_ in terms of *λ*_*i*_, and the margin here can be regarded as a measure for the misclassification degree. If *λ*_*i*_ > 0, then *J* ^−^(*ϕ*_*i*_) > *J* ^+^(*ϕ*_*i*_), and samples may tend to be correctly classified. The larger the value of *λ*_*i*_ (s.t. *λ*_*i*_ > 0) is, the easier the classification is. In practice, we can only select *d* leading eigenvectors to form the transformation matrix Φ corresponding to *d* dominant positive eigenvalues and omit all the eigenvectors with relatively small eigenvalues. In this way, a low-dimensional subspace can be obtained. As reported above in our experiments, MNMDP really achieves the best classification with low dimensionality. If *λ*_*i*_ ≤ 0, then *J* ^−^(*ϕ*_*i*_) < *J* ^+^(*ϕ*_*i*_), and samples may be put into wrong classes. As a consequence, we discard those eigenvectors in respect to nonpositive eigenvalues. It should be noted that the classification performance is seriously degraded when all eigenvalues are nonpositive. In this case, it is difficult to distinguish interclass and intraclass points along the projections. To solve the problem, we can map data points into Hilbert space with some kernel tricks. However, this is out of the scope of this paper and will be discussed in the future work.

**Figure 12:**
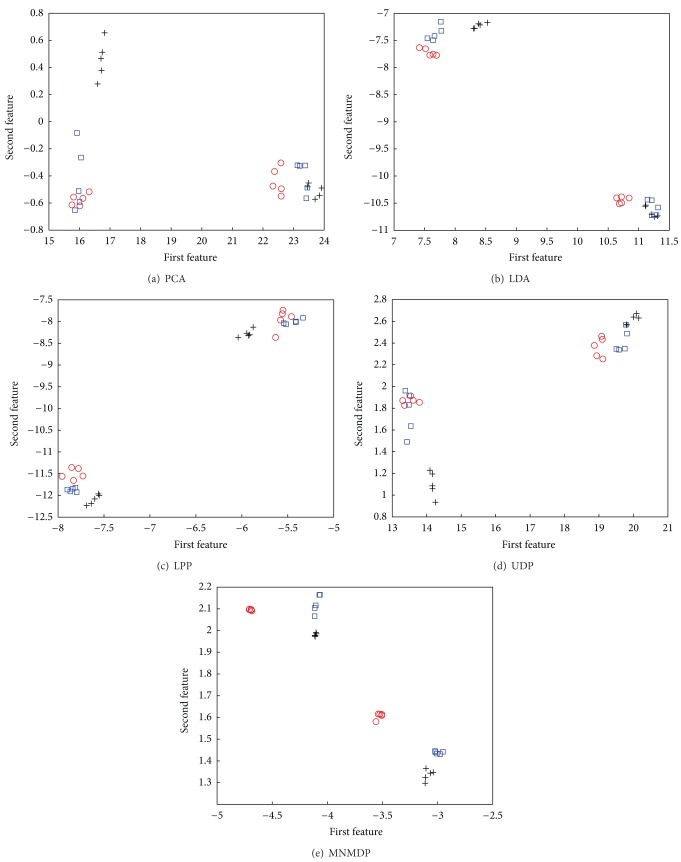
Data distribution of each method in two-dimensional projection space, where the three classes are denoted by “+", “⚪", and “□", respectively.

Based on the discussions aforementioned, the proposed MNMDP has more discrimination for classification. From the perspective of the classification, the key point is to enhance the pattern discrimination between samples from different classes of data. For the high-dimensional data, the good way is to find a projection that makes the samples from the same class compact and ones from different classes separable in the low-dimensional space. To visually verify the superiority of MNMDP for classification, we project the sample images in HRF database (described in Section 4.1) onto a two-dimensional subspace with the competing methods, and the samples from the first three subjects of HRF in the projected space with each method are illustrated in Figure 12. Note that, since the samples of each subject are collected in two separated sessions with different variations, each subject is clustered into two subclasses with each method. We can obviously observe that the separability of class clusters of MNMDP is much better than that of PCA, LDA, LPP, and UDP and the subclasses in MNMDP become more compact. Moreover, there is no overlap between three classes in MNMDP, compared to the other methods. Figure 12 intuitively validates the good discriminating capability of MNMDP for classification. The results are consistent with the observation from the experiments in Section 4 and the analysis in this section.

## 6. Conclusions

In this paper, the MNMDP introduced is a liner supervised dimensionality reduction technique, which can well preserve the local geometric structures of data and fully use class information for classification. In the proposed MNMDP, we employ distinct affinity weight for both intraclass and interclass neighbors of all points and then keep the margin between intraclass and interclass neighborhoods maximum through a liner mapping. In order to well investigate the classification performance of the MNMDP, our experiments are conducted on four high-dimensional databases, in comparison with the competing methods: LPP, UDP, PCA, and LDA. Through the comprehensive experiments, it demonstrates the effectiveness and robustness of the proposed MNMDP with satisfactory performance in pattern classification. In the future work, we plan to extend the MNMDP with some kernel tricks.

## Figures and Tables

**Algorithm 1:**
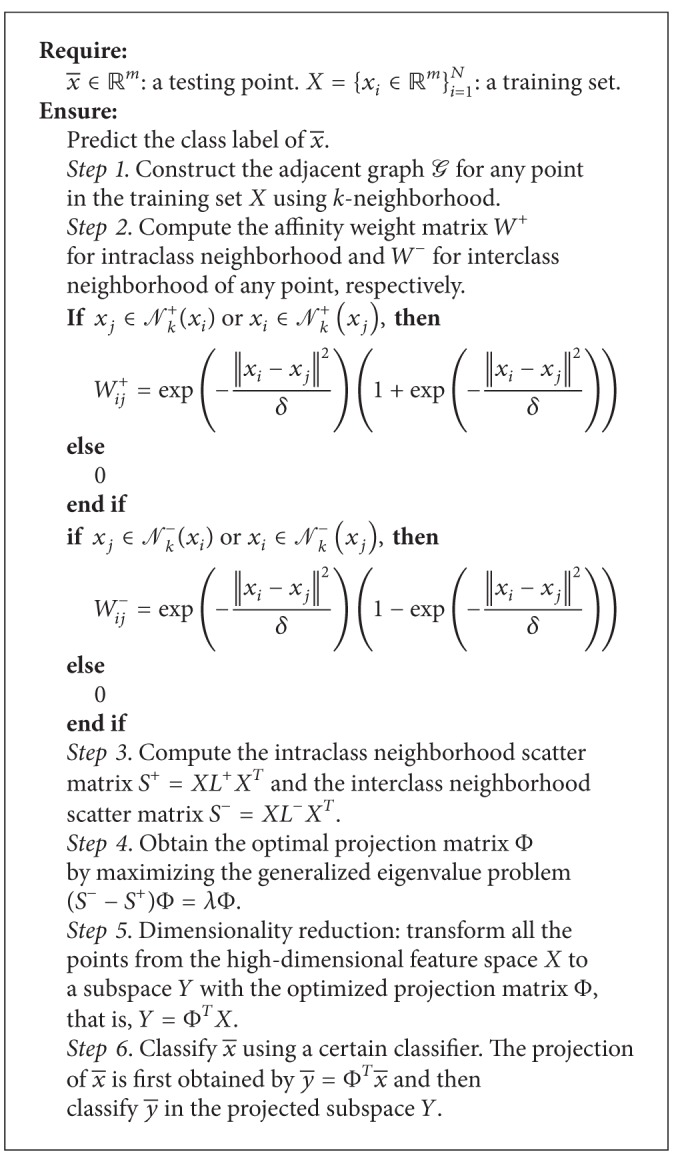
Maximum neighborhood margin discriminant projection.

**Table 1:** The maximal average recognition rates (%) of each method on HRF with the corresponding standard deviations (stds) and values of dimension in the parentheses.

Methods	*l* = 5	*l* = 6	*l* = 7	*l* = 8
PCA	66.96 ± 1.49	71.37 ± 1.27	73.87 ± 1.44	76.99 ± 2.43
	(60)	(55)	(65)	(50)
LDA	68.64 ± 1.31	73.00 ± 1.06	75.43 ± 1.29	78.55 ± 2.31
	(55)	(45)	(40)	(55)
LPP	67.21 ± 1.41	71.58 ± 1.25	74.29 ± 1.09	77.55 ± 2.01
	(135)	(80)	(85)	(65)
UDP	67.41 ± 1.58	71.21 ± 1.26	73.36 ± 1.52	75.57 ± 2.28
	(135)	(145)	(150)	(150)
MNMDP	**74.31** ± **1.62**	**78.47** ± **1.61**	**81.21** ± **1.64**	**83.78** ± **1.58**
	**(30)**	**(30)**	**(30) **	**(40)**

**Table 2:** The maximal average recognition rates (%) of each method on FKP with the corresponding standard deviations (stds) and values of dimension in the parentheses.

Methods	*l* = 5	*l* = 7	*l* = 9	*l* = 11
PCA	63.31 ± 2.52	76.56 ± 2.80	85.18 ± 1.98	90.75 ± 2.40
	(110)	(125)	(130)	(110)
LDA	65.80 ± 2.35	79.44 ± 2.88	88.02 ± 2.01	92.80 ± 2.07
	(105)	(115)	(125)	(125)
LPP	64.30 ± 2.43	78.36 ± 2.52	86.80 ± 1.80	91.50 ± 2.31
	(110)	(110)	(120)	(105)
UDP	64.88 ± 2.32	78.16 ± 2.84	86.37 ± 1.83	91.30 ± 2.45
	(110)	(120)	(125)	(135)
MNMDP	**69.49** ± **1.98**	**82.96** ± **2.54**	**90.98** ± **1.59**	**94.45** ± **1.90**
	**(70)**	**(50) **	**(55)**	**(65)**

**Table 3:** The maximal average recognition rates (%) of each method on AR with the corresponding standard deviations (stds) and values of dimension in the parentheses.

Methods	*l* = 7	*l* = 9	*l* = 11	*l* = 13
PCA	64.17 ± 2.05	71.60 ± 1.40	76.70 ± 3.28	81.80 ± 3.46
	(100)	(100)	(110)	(100)
LDA	70.94 ± 1.74	78.24 ± 1.09	83.80 ± 2.82	88.80 ± 2.04
	(60)	(55)	(55)	(45)
LPP	65.23 ± 1.79	72.60 ± 1.02	78.20 ± 2.67	83.10 ± 3.31
	(85)	(95)	(80)	(95)
UDP	65.27 ± 1.68	72.22 ± 0.96	77.43 ± 3.17	82.80 ± 4.18
	(100)	(105)	(105)	(105)
MNMDP	**81.63** ± **3.24**	**89.52** ± **2.99**	**94.37** ± **2.26**	**99.00** ± **0.94**
	**(120)**	**(125)**	**(140)**	**(120)**

**Table 4:** The maximal average recognition rates (%) of each method on Musk with the corresponding standard deviations (stds) and values of dimension in the parentheses.

Methods	*l* = 50	*l* = 80	*l* = 110	*l* = 140
PCA	77.82 ± 2.85	79.21 ± 2.36	81.05 ± 2.47	83.72 ± 3.2
	(33)	(40)	(42)	(19)
LDA	77.87 ± 2.72	79.59 ± 3.08	81.25 ± 2.25	83.47 ± 3.45
	(35)	(38)	(40)	(43)
LPP	77.66 ± 2.88	80.28 ± 2.45	81.45 ± 2.72	85.56 ± 3.17
	(33)	(24)	(32)	(25)
UDP	77.63 ± 3.37	79.81 ± 2.63	80.94 ± 2.31	84.18 ± 3.83
	(33)	(38)	(42)	(24)
MNMDP	**78.03** ± **3.50**	**81.46** ± **2.87**	**85.47** ± **2.25**	**87.91** ± **1.74**
	**(11)**	**(18)**	**(8)**	**(7)**
